# Extracellular Vesicle Levels of Nervous System Injury Biomarkers in Critically Ill Trauma Patients with and without Traumatic Brain Injury

**DOI:** 10.1089/neur.2022.0058

**Published:** 2022-12-19

**Authors:** Vivian A. Guedes, Sara Mithani, Cydni Williams, Dilorom Sass, Ethan G. Smith, Rany Vorn, Chelsea Wagner, Chen Lai, Jessica Gill, Holly E. Hinson

**Affiliations:** ^1^ National Institute of Nursing Research, National Institutes of Health, Bethesda, Maryland, USA.; ^2^ School of Nursing, The University of Texas Health Science Center at San Antonio, San Antonio, Texas, USA.; ^3^ Department of Pediatrics, Oregon Health & Science University, Portland, Oregon, USA.; ^4^ School of Nursing, Johns Hopkins University, Baltimore, Maryland, USA.; ^5^ Department of Neurology, Oregon Health & Science University, Portland, Oregon, USA.

**Keywords:** biomarkers, head trauma, traumatic brain injury

## Abstract

Moderate/severe traumatic brain injury (TBI) causes injury patterns with heterogeneous pathology producing varying outcomes for recovery. Extracellular vesicles (EVs) are particles containing a myriad of molecules involved in cell signaling. EVs may hold promise as biomarkers in TBI because of their encapsulation, including improved stability/decreased degradation. A subset of subjects with and without TBI from a prospective, observational trial of critically ill trauma patients were analyzed. Total EV levels of glial (glial fibrillary acidic protein; GFAP) and neuronal/axonal (ubiquitin carboxy-terminal hydrolase L1 [UCH-L1], neurofilament light chain [NfL], and total-tau) proteins were measured using single-molecule array technology. Protein levels were winsorized to address outliers and log transformed for analysis. Patients with multiple injuries (*n* = 41) and isolated body injury (*n* = 73) were of similar age and sex. Patients with multiple injuries were, as expected, more severely injured with higher Injury Severity Scores (29 [26–41] vs. 21 [14–26], *p* < 0.001) and lower Glasgow Coma Scale scores (12 [4–13] vs. 13 [13–13], *p* < 0.001). Total body EVs of GFAP, UCH-L1, and NfL were higher in those with multiple injuries (1768 [932–4780] vs. 239 [63–589], *p* < 0.001; 75.4 [47.8–158.3] vs. 41.5 [21.5–67.1], *p* = 0.03; 7.5 [3.3–12.3] vs. 2.9 [2.1–4.8], *p* < 0.001, respectively). There was a moderate correlation between the Head Abbreviated Injury Score and GFAP (free circulating rho = 0.62, EV rho = 0.64; both *p* < 0.001). Brain-derived proteins contained in EV holds promise as an informative approach to biomarker measurement after TBI in hospitalized patients. Future evaluation and longitudinal studies are necessary to draw conclusions regarding the clinical utility of these biomarkers.

## Introduction

Moderate/severe traumatic brain injury (TBI) encompasses a set of heterogeneous injury patterns with potentially devasting consequences. To better navigate this heterogeneity, investigators have pursued methods to better endophenotype patients, especially with blood-based biomarkers. Protein markers of nervous system injury appear especially promising, correlating well with injury severity^1,2^ and long-term outcome.^3^

Circulating protein biomarkers are most often quantified by measuring free or unbound proteins in the fluid of interest (e.g., blood, cerebrospinal fluid). However, markers of interest may also be encapsulated in extracellular vesicles (EVs). EVs are composed of a lipid bilayer envelope and contain cargo-like proteins, lipids, and RNA,^4,5^ which can be used for cell-to-cell communication.^6,7^ EV subtypes include exosomes, microvesicles (also described as microparticles), and apoptotic bodies.^8,9^ EVs have been linked to pathological processes, but mechanisms involving EV-based cell-to-cell communication in health and disease are only beginning to be understood.^6,10^ There are some potential benefits of measuring EV encapsulated biomarkers, including improved stability/decreased degradation in circulation,^11^ the ability to cross the blood–brain barrier,^12^ and the ability to determine the cell type from which the biomarker originated.^5,13^ Moreover, not all biologically active molecules can be measured in a soluble form.^14^

Earlier efforts in moderate-to-severe TBI indicate that EV levels of neuronal and glial-injury markers correlate with free circulating levels; however, there also appear to be informative differences between them. EV levels of neurofilament light chain (NfL), a neurofilament protein and marker of axonal injury or degeneration, and glial fibrillary acidic protein (GFAP), a filament protein found in astrocytes, were higher in patients with diffuse brain injury than those with focal injury.^15^

Given that this line of inquiry has recently emerged, a number of questions have yet to be answered.

Studies investigating the utility of central nervous system (CNS) injury-associated proteins have often used healthy controls or small groups of orthopedic controls for comparisons. Levels of neuronal or glial injury proteins are inadequately characterized in patients sustaining major trauma without TBI in the literature. Moreover, to our knowledge, no study has compared EV levels of proteins in major trauma patients with and without TBI.

In order to address some of these gaps, we examined a cohort of subjects who sustained major trauma, both with and without radiographic TBI. We hypothesized that patients with TBI would have higher levels of CNS injury-associated proteins in the circulation and encased in EVs. Further, we also hypothesized that EV levels of each biomarker might be more strongly correlated with TBI severity, as measured by the Head Abbreviated Injury Score (HAIS), than plasma levels.

## Methods

### Subjects

A subset of subjects from the FAINT (Fever And Inflammation in NeuroTrauma) study were analyzed. The details have been previously published,^16^ but, in brief: FAINT was a prospective, observational trial including all adults (age ≥18) suffering from major trauma requiring admission to the intensive care unit. For this analysis, we included subjects with multiple injuries (HAIS score >2, one other region >2) and isolated body injury (one region >2, excluding head/face) based on Abbreviated Injury Severity (AIS) scores. The AIS is an anatomically based injury severity scoring system that classifies each injury by body region on a 6-point scale from no injury (AIS = 0) to maximal/unsurvivable (AIS = 6). The AIS is the system used to determine the Injury Severity Score (ISS) of the multiply injured patient.^17^ It is calculated upon discharge and accounts for overall severity and trajectory of injuries. In the case of the HAIS, information from multiple head computed tomography (CT) scans, if present, is aggregated. Per convention, TBI was defined as HAIS >2 in this investigation. Admission blood samples were obtained within 6–12 h of the antecedent trauma, centrifuged for plasma, and stored at −80°C for batch analysis. Institutional review board approval was obtained before first enrollment, and informed consent was obtained from each participant or their legally authorized representative.

### Isolation of extracellular vesicles from plasma

Total body EVs (including microvesicles, exosomes, ectosomes, and others) were isolated from 400 μL of frozen human plasma containing ethylene diamine tetraacetic acid. After thaw, plasma samples were centrifuged at 3000*g* for 15 min at 4°C twice to remove cells and cell debris. Samples were then transferred to a clean tube for EV isolation. EVs were precipitated by using ExoQuick™ Plasma Prep and the Exosome Precipitation Kit (EXOQ5TM-1; System Biosciences Inc., Mountain View, CA), according to the manufacturer's instructions. After adding ExoQuick to each sample, the mixture was incubated for 1 h at 4°C. Samples were kept upright during incubation and subsequently centrifuged at 1500*g* for 30 min. After the centrifugation, EVs appeared as pellets at the bottom of the tube. The supernatant was aspirated from each tube, and each pellet was resuspended in 200 μL of Dulbecco's phosphate-buffered saline (14190144; ThermoFisherScientific, Waltham, MA). Samples were then stored at −80°C until analysis. For protein quantification, each tube received equal sample volumes of lysis buffer (M-PER^TM^ mammalian protein extraction reagent; 78501; ThermoFisherScientific), containing a protease inhibitor cocktail (cOmplete™ ULTRA Tablets; MilliporeSigma, Burlington, MA). These suspensions were used to measure protein concentrations. For particle characterization, EV samples were analyzed using the MACSPlex Exosome Kit to access the presence of specific EV membrane markers (130-108-813; Miltenyi Biotec, Bergisch Gladbach, Germany) and an nCS1 particle-size analyzer to measure the approximate size and concentrations of EVs (Spectradyne, Signal Hill, CA).

### Protein analysis

EV and plasma levels of GFAP, ubiquitin carboxy-terminal hydrolase L1 (UCH-L1), NfL, and total-tau proteins were measured using an ultra-sensitive paramagnetic bead-based enzyme-linked immunosorbent assay (Neuro 4-plex A kit; 102153; Quanterix, Lexington, MA) in a site-specific single-molecule array (Simoa) HD-1 analyzer (Quanterix), according to the manufacturer's protocol. Simoa technology has been widely used for quantification of blood-based biomarkers. The assay provided by the manufacturer includes eight calibrators to generate standard curves and two positive controls. Each sample was measured in duplicate by investigators blinded to clinical groups and the average result reported. All samples were analyzed in the same experiment, and coefficients of variation (CVs) for each sample were calculated. The accepted intraplate coefficients of CVs of analyzed samples were no higher than 20% for all analytes.

### Statistical analysis

Total EV levels of GFAP and tau levels were winsorized to address outliers. Levels of all plasma biomarkers were winsorized to address outliers. Measurements for all biomarkers were log transformed for analysis. Group comparisons between multiple injuries and isolated body trauma were made with *t*-tests on continuous variables; categorical variables were compared with chi-squared tests. The CV was calculated by dividing the standard deviation of the protein of interest by the mean of the same protein and multiplying the result by 100 (expressed as a percentage). Correlations between plasma and EV biomarker levels were measured with a Pearson's correlation coefficient on winsorized, log-transformed values. All analyses were performed in R software (4.0.3; R Core Team 2020). Significance was set at *p* < 0.05.

## Results

### Subjects and injury characteristics

One hundred fourteen subjects from the FAINT data repository were analyzed, including those with multiple injuries (brain and body injury, *n* = 41) and those with isolated body injury (*n* = 73). The were no differences between injury groups with respect to age, sex, or racial/ethnic identity. As expected, based on group definition, subjects with multiple injuries had significantly higher ISSs (29 [26–41] vs. 21 [14–26], *p* < 0.001), higher HAIS (4 [3–4] vs. 0 [0–0], *p* < 0.001), and lower admission Glasgow Coma Scale (GCS) scores (12 [4-13] vs. 13 [13–13], *p* < 0.001). (Table 1; Supplementary Fig. 1).

**FIG. 1.**
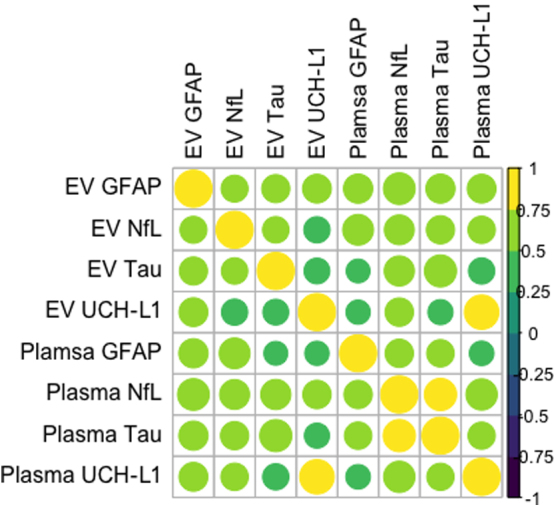
Correlation matrix showing the relationships between EV and free plasma levels of each marker. Free circulating and EV levels were highly, significantly correlated with each other (*r* = 0.83–0.96, *p* < 0.001). EV, extracellular vesicle; GFAP, glial fibrillary acidic protein; NfL, neurofilament light chain; UCH-L1, ubiquitin carboxy-terminal hydrolase L1.

**Table 1.** Demographics and Injury Severity of the Participants

Characteristic	Overall,* N* = 114^a^	Multiple injuries,* N* = 41^a^	Isolated body injury,* N* = 73^a^	*p* value^b^
Age, years	49 (31, 59)	51 (32, 59)	46 (31, 60)	>0.9
Sex (%)				0.7
Male	83 (73)	29 (71)	54 (74)	
Female	31 (27)	12 (29)	19 (26)	
Race and ethnicity (%)				0.2
White	98 (88)	38 (97)	60 (83)	
Black or African American	3 (2.7)	0 (0)	3 (4.2)	
Asian	0 (0)	0 (0)	0 (0)	
Hispanic	7 (6.3)	1 (2.6)	6 (8.3)	
Other	3 (2.7)	0 (0)	3 (4.2)	
Unknown	3	2	1	
ISS	23 (17, 30)	29 (26, 41)	21 (14, 26)	**<0.001**
Admission GCS score	13 (12, 13)	12 (4, 13)	13 (13, 13)	**<0.001**
HAIS (%)				**<0.001**
0	62 (54)	0 (0)	62 (85)	
1	2 (1.8)	0 (0)	2 (2.7)	
2	9 (7.9)	0 (0)	9 (12)	
3	19 (17)	19 (46)	0 (0)	
4	13 (11)	13 (32)	0 (0)	
5	9 (7.9)	9 (22)	0 (0)	

Groups were similar in age, sex, and racial/ethnic identity. Those with multiple injuries were more severely injured than the isolated body group.

^a^ Median (IQR); *n* (%).

^b^ Wilcoxon's rank-sum test; Pearson's chi-squared test; Fisher's exact test.

ISS, Injury Severity Score; GCS, Glasgow Coma Scale; HAIS, Head Abbreviated Injury Score; IQR, interquartile range.

### Free circulating and extracellular vesicle central nervous system injury-associated protein levels

Total body EV levels of injury-associated proteins were strongly and significantly correlated to free circulating plasma levels of each respective marker: GFAP (*r* = 0.96, *p* < 0.001; confidence interval [CI], 0.94–0.97); tau (*r* = 0.88, *p* < 0.001; CI, 0.83–0.92); NfL (*r* = 0.85, *p* < 0.001; CI, 0.79–0.90); and UCH-L1 (*r* = 0.83, *p* < 0.001; CI, 0.76–0.88; Fig. 1). Only weak correlations (*r* < 0.50) existed between free circulating or EV levels of each protein and total ISS.

We quantified the CV of each marker by EV levels and free-circulating plasma level, and found that CVs indicated moderate-to-high dispersion for each marker. GFAP was most similar between EV and plasma levels; the EV CV was 146% vs. 142% in plasma. In contrast, UCH-L1 was more variable when EV levels were measured (140% vs. 86%), as was NfL, EV (130%), and plasma (74%). Finally, tau displayed much lower variability when measured in EV levels (94% vs. 244%).

### Extracellular vesicle levels of injury-associated proteins were higher in patients with traumatic brain injury

**FIG. 2.**
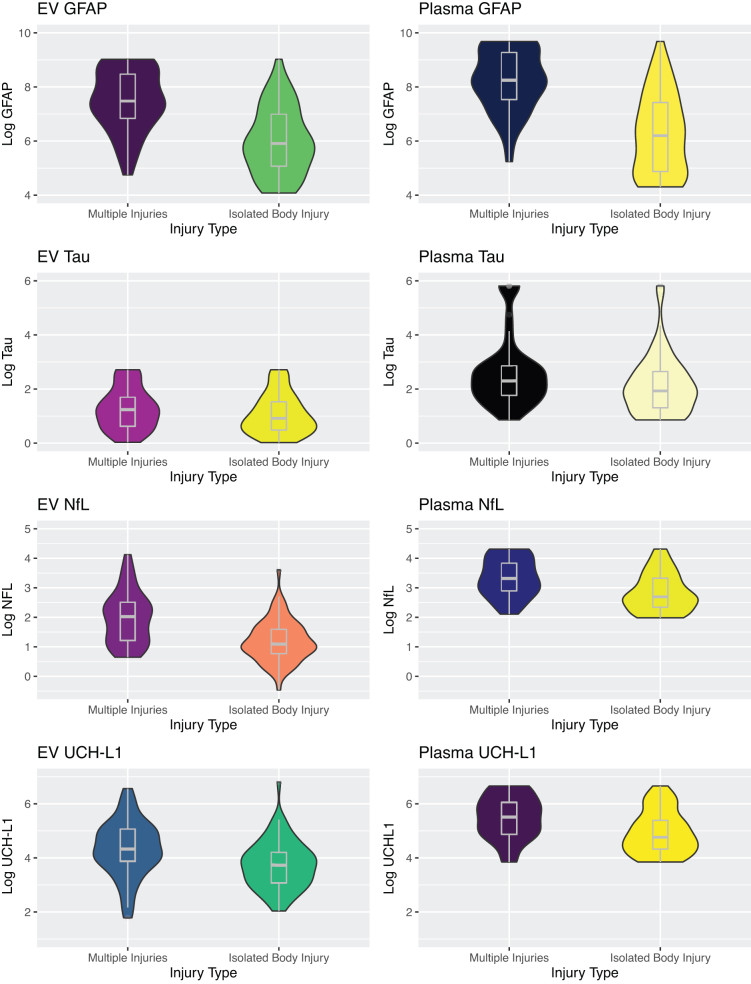
Violin plots of each marker by injury type demonstrating distributions. Median levels of GFAP, NfL, and UCH-L1 were different between groups (*p ≤ 0.05). GFAP, glial fibrillary acidic protein; NfL, neurofilament light chain; UCH-L1, ubiquitin carboxy-terminal hydrolase L1.

Total body EVs of GFAP, UCH-L1, and NfL were higher in those with multiple injuries than those without clinical evidence of TBI (1768 [932–4780] vs. 239 [63–589] pg/mL, *p* < 0.001; 75.4 [47.8–158.3] vs. 41.5 [21.5–67.1] pg/mL, *p* = 0.03; 7.5 [3.3–12.3] vs. 2.9 [2.1–4.8] pg/mL, *p* < 0.001, respectively). There were no significant differences in EV tau levels. Similarly, free circulating levels of GFAP, UCH-L1, and NfL were higher in those with multiple injuries than those without clinical evidence of TBI (3816 [1866-10,642] vs. 493.1 [130.7–1679.0] pg/mL, *p* < 0.001; 246.3 [130.4–421.5] vs. 117 [75.6–218.8] pg/mL, *p* < 0.001; 27.3 [18.1–46.2] vs. 14.8 [10.4–27.8] pg/mL, *p* < 0.001, respectively). There were no significant differences in plasma tau levels (Fig. 2).

### Extracellular vesicle versus plasma injury-associated protein levels by severity of traumatic brain injury

**FIG. 3.**
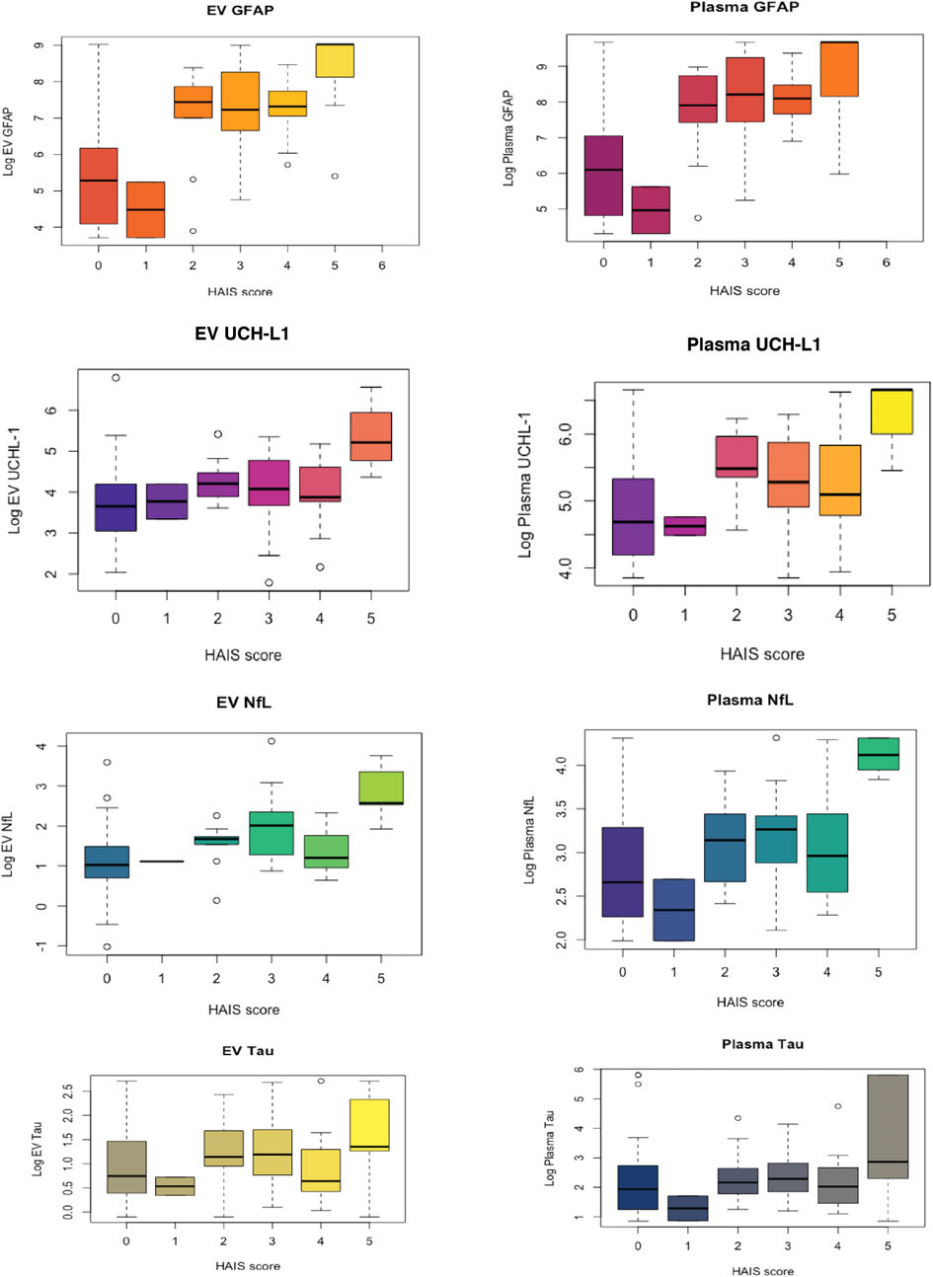
Box plots of each marker by severity of TBI (HAIS). GFAP, glial fibrillary acidic protein; HAIS, Head Abbreviated Injury Score; NfL, neurofilament light chain; UCH-L1, ubiquitin carboxy-terminal hydrolase L1.

To assess the relationship between severity of TBI and biomarker levels, patients were divided into groups based on the HAIS (0–5). Median levels of each protein were visualized both in EVs and plasma to assess patterns as clinical severity of injury increased and whether patterns differed in measurement method (plasma vs. EV). Generally, as HAIS increased, median levels of each marker increased, regardless of where it was measured. Additionally, there appears to be a clustering of lower levels in patients with HAIS of 0 or 1 and higher levels in patients with HAIS 2–5. Because subgroups were very small, formal significance was not assessed (Fig. 3).

## Discussion

In this investigation, we found that total body EV levels of the CNS injury-associated proteins GFAP, UCH-L1, and NfL, but not tau, are higher in hospitalized multiple trauma patients with radiographical moderate-severe TBI when compared to patients with multiple injuries without TBI. To our knowledge, this is the first study to compare EV levels of neuronal and glia injury markers in multiple trauma patients with and without TBI. We also observed that EV levels of all four proteins were strongly and significantly correlated to free circulating levels in plasma shortly after injury. We also noticed some informative patterns. For example, GFAP was the most highly correlated between EV levels and free circulating plasma levels. Additionally, in some cases, the method of measure (EV vs. plasma) produced differences in the dispersion of results (e.g., UCH-L1, NfL, and tau), which might influence sample-size planning for future studies.

Our work has several potential implications. First, our results suggest that free circulating plasma levels of several of the injury-associated proteins might be adequate to ascertain the burden of neurological injury immediately after injury in severely injured patients. This is logical, given that our sampling occurred at a time (hours after injury) when the blood–brain barrier was likely maximally open, as has been observed in experimental models of TBI.^18^ Still, measurement of EVs may provide a wealth of information depending on the research question and the timing of the measurement.

Other investigators have observed injury-specific differences in free circulating versus exosomal levels of injury-associated proteins. For example, Mondello and colleagues observed that patients with diffuse injury displayed higher acute EV NfL and GFAP concentrations than those with focal lesions in another cohort.^15^ Acute levels of EV versus plasma proteins could be informative regarding long-term outcomes. In this case, longitudinal studies evaluating neurobehavioral outcomes are warranted to evaluate the prognostic potential of EVs in comparison to plasma proteins. Our group previously reported that EV NfL levels are more tightly correlated with the severity of post-concussive symptoms than plasma protein levels.^19^ Thus, severity of injury and time course, as well as outcome of interest, will likely dictate the most appropriate approach to biomarker measurement.

Contrary to our hypothesis, we also noted weak correlations between clinical scales of injury severity (ISS, HAIS) and levels of biomarkers in EVs and plasma without significant differences in the method of measurement. There are several potential explanations for this. It could be that peripheral nervous system injury contributes to the higher levels measured in peripheral blood (both free circulating and in EVs), giving the impression that more CNS damage was present than which truly occurred. Other tissues can be reservoirs of these proteins, despite the fact that the CNS holds the highest concentration of GFAP^20^ and tau.^21^

It is also possible that our clinical scales of severity are imprecise and poorly classify the true severity of brain injury. Clinical scales such as the GCS are notoriously poor at injury phenotyping TBI.^22^ Some researchers have asserted that anatomically based injury scales (e.g., ISS and HAIS) might appear to capture injury better^23^ and be less prone to pollution by intoxication or other confounders; they might still be less than ideal for capturing CNS injury. Classification systems like the HAIS based on CT scans may miss important intracranial lesions, including axonal injury, leading to the misclassification of some patients with clinically important CNS injury. Support for this hypothesis comes from literature showing that magnetic resonance imaging is more sensitive than CT for these particular lesions in TBI,^24^ as well as our data showing GCS <15 in patients with HAIS scores of 0. Patients with concussion can have clinical evidence of brain injury and elevation of neuronal injury biomarkers without radiographical abnormalities on head imaging.^25^ Patients with significant body trauma requiring hospitalization are likely to have injury mechanisms placing them at high risk for concussion. Future studies might classify HAIS of >1 in the category of TBI as opposed to >2 as we defined it in this study based on the observed distinction in median biomarker levels at this less severe presentation.

EVs are increasingly gaining attention in TBI for their unique ability to shield and preserve signals from the injured brain, allowing for the measurement of biomarkers that were previously difficult to detect and lending important physiological insights. Recently, investigators showed that traumatically injured neurons release osteogenic microRNA-enriched EVs, which target osteoprogenitors in bone to stimulate bone formation in both animals and humans.^26^ In mild TBI (mTBI), EV levels of biomarkers have tracked more closely with symptoms; for example, higher levels of tau, amyloid-beta 42, and interleukin-10 have been associated with mTBI and chronic symptoms in military personnel.^27^ Investigators from the University of Pennsylvania compared a cohort of mTBI with healthy controls and orthopedic patients, and found that GluR2^+^ EVs had distinct biomarker distributions than those present in plasma. However, in contrast to our observations, they found that brain-derived proteins (GFAP, NfL, Tau, and UCH-L1) correlated more with each other within the same compartment than between the plasma and EV compartments.^28^ A potential source of this difference may be attributable to our ultra-early time point and our more severely injured population. It could be that important differences in plasma and EV levels of brain-derived proteins emerge with time, as the work of Mondello and colleagues suggests,^15^ attributable to lower degradation of EV encapsulated proteins over time and/or closure of the blood–brain barrier. Before harnessing EVs for innovative applications such as prognostication, monitoring therapeutic response, or even drug delivery to the CNS,^29^ better characterization of the natural history of cell-specific EV contents and expression time course is essential.

### Limitations

There are a number of important limitations to our work. Patients classified as isolated body injury might have experienced subclinical TBI, or even concussion, which is suggested by the levels of CNS injury markers we observed. It is unclear what influence CNS injury versus sedation and analgesia had on admission GCS scores, the median of which was below normal at 13 in the isolated body group. Our analysis focused on a very early time point after injury to capture the initial injury with minimal influence of secondary brain injury. Given that we did not analyze longitudinal data, our results might not be representative of later time points or trajectories. Several markers' peak expression is later than the first 8 h after injury, so it is possible that other patterns might emerge at 24–48 h not noted here.

## Conclusion

Measurement of brain-derived proteins contained in EVs holds promise as a potentially informative approach to biomarker measurement after TBI in hospitalized trauma patients. However, the patient population, time point after injury, and protein in question modify the utility of measuring EV levels of biomarker over free-circulating plasma levels. Measurement of total levels of brain-derived proteins (GFAP, NfL, Tau, and UCH-L1) might be unnecessary in the hyperacute phase after moderate-severe TBI, though more research is needed on the discriminate ability of EV biomarkers over plasma in detecting CNS injury. Future work exploring CNS cell-specific EV levels of biomarkers (e.g., inflammatory cytokines, microRNA, and messenger RNA) is of potential interest, and longitudinal studies would be necessary to draw conclusions regarding the clinical utility of biomarkers evaluated in the study.

## Supplementary Material

Supplemental data

Supplemental data

Supplemental data

## Abbreviations Used

CI: confidence interval
CNS: central nervous system
CT: computed tomography
CVs: coefficients of variation
EVs: extracellular vesicles
GCS: Glasgow Coma Scale
GFAP: glial fibrillary acidic protein
HAIS: Head Abbreviated Injury Score
ISS: Injury Severity Score
mTBI: mild TBI
NfL: neurofilament light chain
Simoa: single-molecule array
TBI: traumatic brain injury
UCH-L1: ubiquitin carboxy-terminal hydrolase L1
