# Supplementary material for: Extracellular Vesicle Levels of Nervous System Injury Biomarkers in Critically Ill Trauma Patients with and without Traumatic Brain Injury
Source: Neurotrauma Rep. 2022 Dec 19;3(1):545–53. doi: 10.1089/neur.2022.0058 (PMC9811954; doi:10.1089/neur.2022.0058)

**Supplemental Figure 1.** Stacked bar graph of the Abbreviated Injury Score of each body system by trauma group.


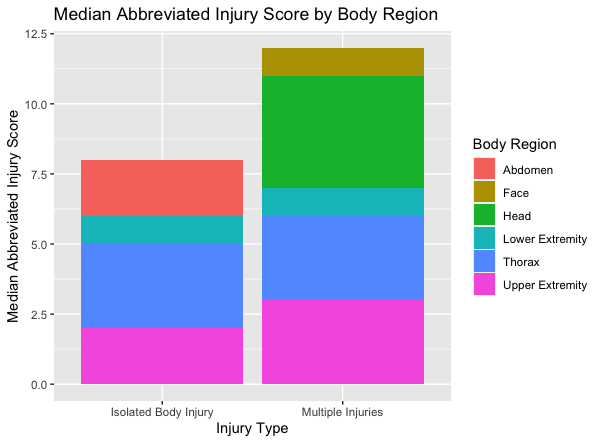

Supplement: Supplemental data [file Suppl_FigS1.docx]
