# Supplementary material for: Extracellular Vesicle Levels of Nervous System Injury Biomarkers in Critically Ill Trauma Patients with and without Traumatic Brain Injury
Source: Neurotrauma Rep. 2022 Dec 19;3(1):545–53. doi: 10.1089/neur.2022.0058 (PMC9811954; doi:10.1089/neur.2022.0058)
Supplement: Supplemental data [file Suppl_FigS2.docx]

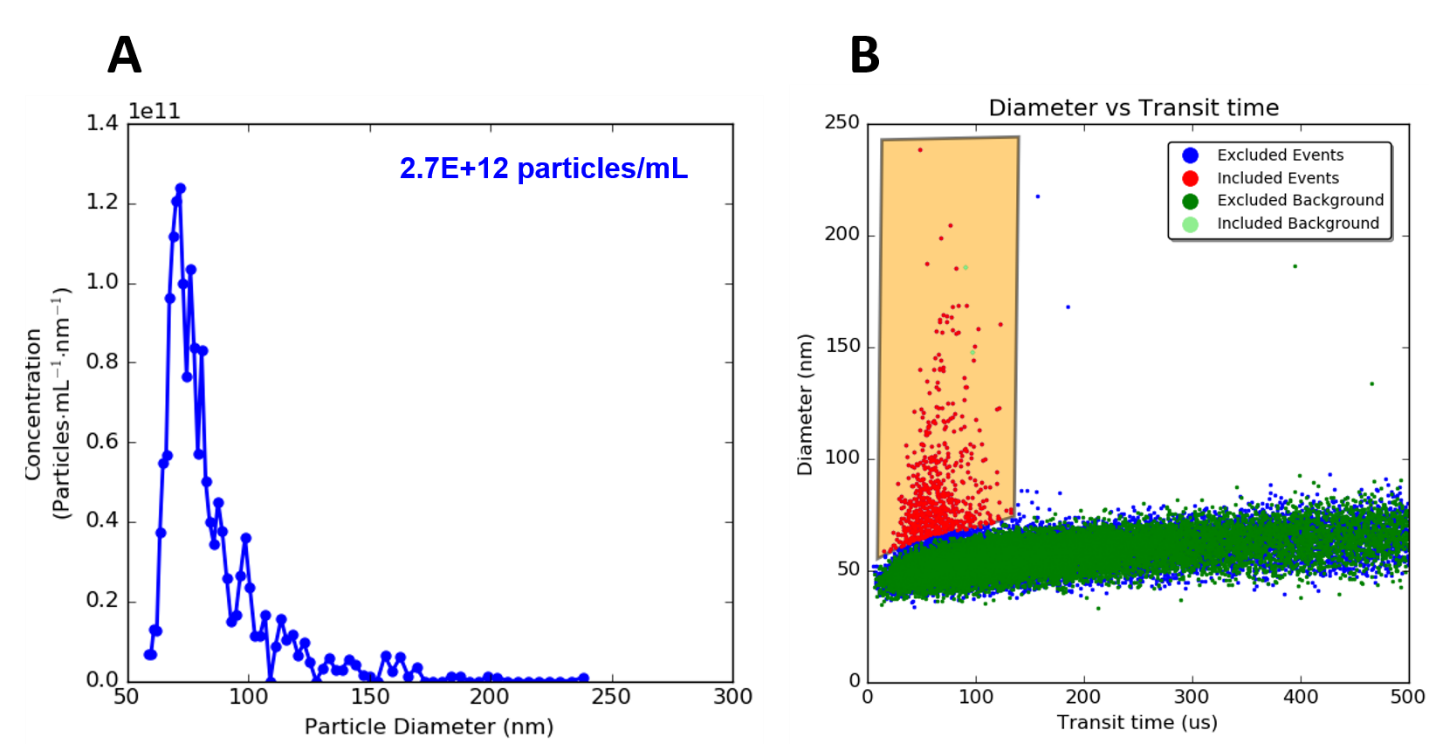


**Supplementary Figure 2: Extracellular vesicle size and concentration.**

We characterized EV size and concentration with microfluidic resistive pulse sensing using a CS1 instrument (hardware version 1, Spectradyne, Signal Hill, CA). **A.** The concentration spectral density plot is shown for a representative EV sample, with the concentration for that sample shown in blue text. The mean particle concentration of EV samples (n = 6) was 3.4 x 10^12^ particles/mL (Standard Deviation [SD] = 1.6 x 10^12^ particles/mL), and the mean peak diameter was 69.2nm (SD = 9.0nm). **B.** Data was analyzed using the Spectradyne Data Viewer software (version 2.5.0.275). To differentiate real particles from background noise, the nCS1 background subtract feature was used; estimated background noise is shown in dark and light green. The peak filtering strategy was to draw a polygon gate around the population of real particles (shown in red) above the green population of background noise centered around 50nm, based on recommendations from the manufacturer. The gate used for this sample is shown in orange. The final concentration and diameter are calculated based on the peak filtering strategy that is applied, and this strategy is representative of how each EV sample was analyzed. C300 cartridges (50nm – 300nm measurement range) were used for all measurements. The instrument specifications are determined automatically based on the cartridge type and the cartridge mold ID. Each sample was diluted in a buffer consisting of 2% tween 20 in 1X PBS filtered at 0.2µm, and the concentration spectral density graph of each sample was adjusted based on the dilution factor of each EV sample. 5uL of the diluted EV sample was loaded onto each C300 cartridge.
