# Supplementary material for: Extracellular Vesicle Levels of Nervous System Injury Biomarkers in Critically Ill Trauma Patients with and without Traumatic Brain Injury
Source: Neurotrauma Rep. 2022 Dec 19;3(1):545–53. doi: 10.1089/neur.2022.0058 (PMC9811954; doi:10.1089/neur.2022.0058)
Supplement: Supplemental data [file Suppl_FigS3.docx]

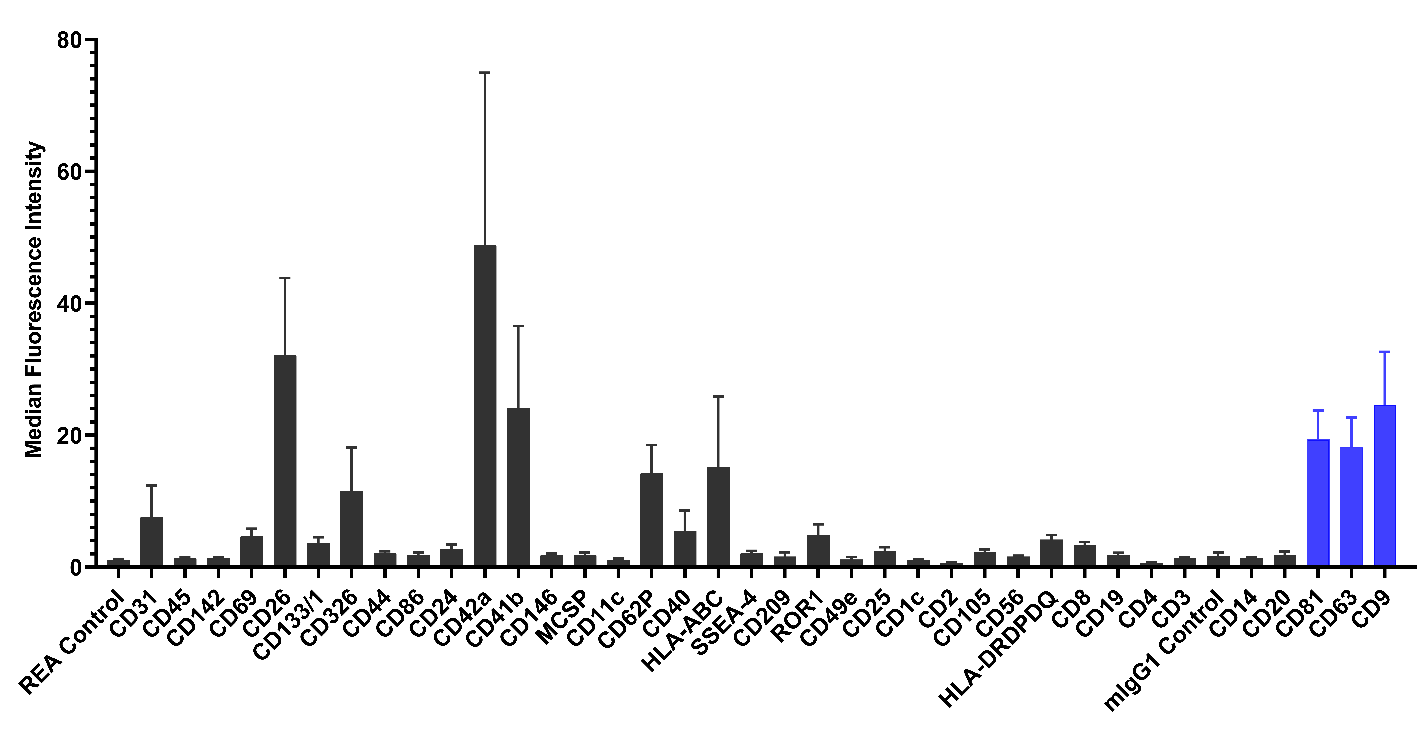


**Supplementary Figure 3**. **Characterization of extracellular vesicles (EVs) using bead-based multiplex flow cytometry.** Signal intensity of each bead population. EV markers CD81, CD63, and CD9 (EV markers) are highlighted (Blue). Controls and other markers that are included in the MACSPlex Exosome Kit (130-108-813, Miltenyi Biotec, Bergisch Gladbach, Germany) are also shown (Black). Flow cytometric analysis was performed with a MACSQuant Analyzer 10 flow cytometer equipped with 405, 488, and 638 nm lasers (Miltenyi Biotec) by using the built-in 96-well plate reader. EV-containing samples were processed according to manufacturer’s instructions. FlowLogic and MACSQuant software (Miltenyi Biotec) were used to analyze flow cytometric data. The median fluorescence intensity (MFI) for all 39 capture bead subsets were background corrected by subtracting respective MFI values from negative control (non-EV buffer + capture beads + antibodies). The error bar represents the standard error of the mean.
